# Supplementary material for: Ecotypes, Wolbachia, and urbanization shape Culex pipiens population structure in a West Nile virus hotspot
Source: bioRxiv. 2026 May 13:2026.05.10.722846. Preprint. [Version 1] doi: 10.64898/2026.05.10.722846 (PMC13192595; doi:10.64898/2026.05.10.722846)
Supplement: Supplement 1 [file media-1.pdf]

Supplementary Methods

Genome annotation

The assembly for *Culex pipiens pipiens* (accession number GCA_963924435.1) was downloaded from NCBI genome database. The seqIDs were replaced by new seqIDs containing the number of the chromosomes using custom Perl one-line (perl -ne) commands (https://github.com/socebrian/pipiens_popgen/blob/main/6.Annotation_and_enrichment.md). A mapping file was created with old and new sequence IDs. The assembly was evaluated with QUAST v5.2.0 (Gurevich et al., 2013) and BUSCO v5.6.1 (diptera_odb10 dataset) (Simão et al., 2015). Repetitive elements were annotated with the EarlGrey pipeline v5.1.1 (Baril et al., 2024) with the default parameters, rDNA elements were annotated with Barrnap v0.9. (Seemann, 2025) and tDNA elements were annotated with tRNAscan-SE v2.0.12 (Chan et al., 2021).

Evidence-based gene structural annotation was performed with transcriptomic and protein sequence data. In brief, 36 transcriptomic datasets (RNA-Seq) were downloaded from EBI database (study ERX10251346). They were processed with Fastq-mcf v1.04.676 (q ≥30, length ≥50 bp) (Aronesty, 2013) and mapped to the reference assembly with STAR v2.7.11b (Dobin et al., 2013), with default parameters compatible with StringTie . We generated transcript models with StringTie v2.2.3 (Pertea et al., 2015) and identified CDS with TransDecoder v5.7.1 (Haas, 2025). We downloaded genome assemblies and annotations for 15 species (GenBank assembly IDs between parenthesis) from NCBI: *Aedes aegypti* (GCF_002204515.2), *Aedes albopictus* (GCF_035046485.1), *Anopheles funestus* (GCF_943734845.2), *Anopheles gambiae* (GCF_943734735.2), *Anopheles stephensi* (GCF_013141755.1), *Armigeres subalbatus* (GCF_024139115.2), *Culex pipiens subsps. pallens* (GCF_016801865.2), *Culex quinquefasciatus* (GCF_015732765.1), *Malaya genurostris* (GCF_037179485.1), *Ochlerotatus camptorhynchus* (GCF_037179485.1), *Sabethes cyaneus* (GCF_943734655.1), *Topomyia yanbarensis* (GCF_030247195.1), *Toxorhynchites rutilus subsp. septentrionalis* (GCF_029784135.1*),* *Uranotaenia lowii* (GCF_029784155.1) and *Wyeomyia smithii* (GCF_029784165.1). We then used these data and GeMoMa v1.9 (Keilwagen et al., 2016) to perform two functional annotations, one using *Culex genomes* and another using all Culicidae data.

We used BRAKER3 (Gabriel et al., 2024) and EviAnn v2.0.2 (Zimin et al., 2025) pipelines for genome annotation. BRAKER was run with RNA-Seq only (Braker1), proteins only (Braker2), and both datasets (Braker3). EviAnn was run with both RNA-Seq and closely related proteins datasets. We selected EviAnn annotation as the best performing annotation after evaluating them with GAQET2 (https://github.com/victorgcb1987/GAQET2, in prep). Functional annotation was performed by sequence homology of the protein sequences identified with EviAnn using

Diamond v2.0.14.152 against SwissProt and TrEMBL datasets (Uniprot, Feb 2025), and curated with AHRD v3.11 (https://github.com/groupschoof/AHRD).

Supplementary Figures

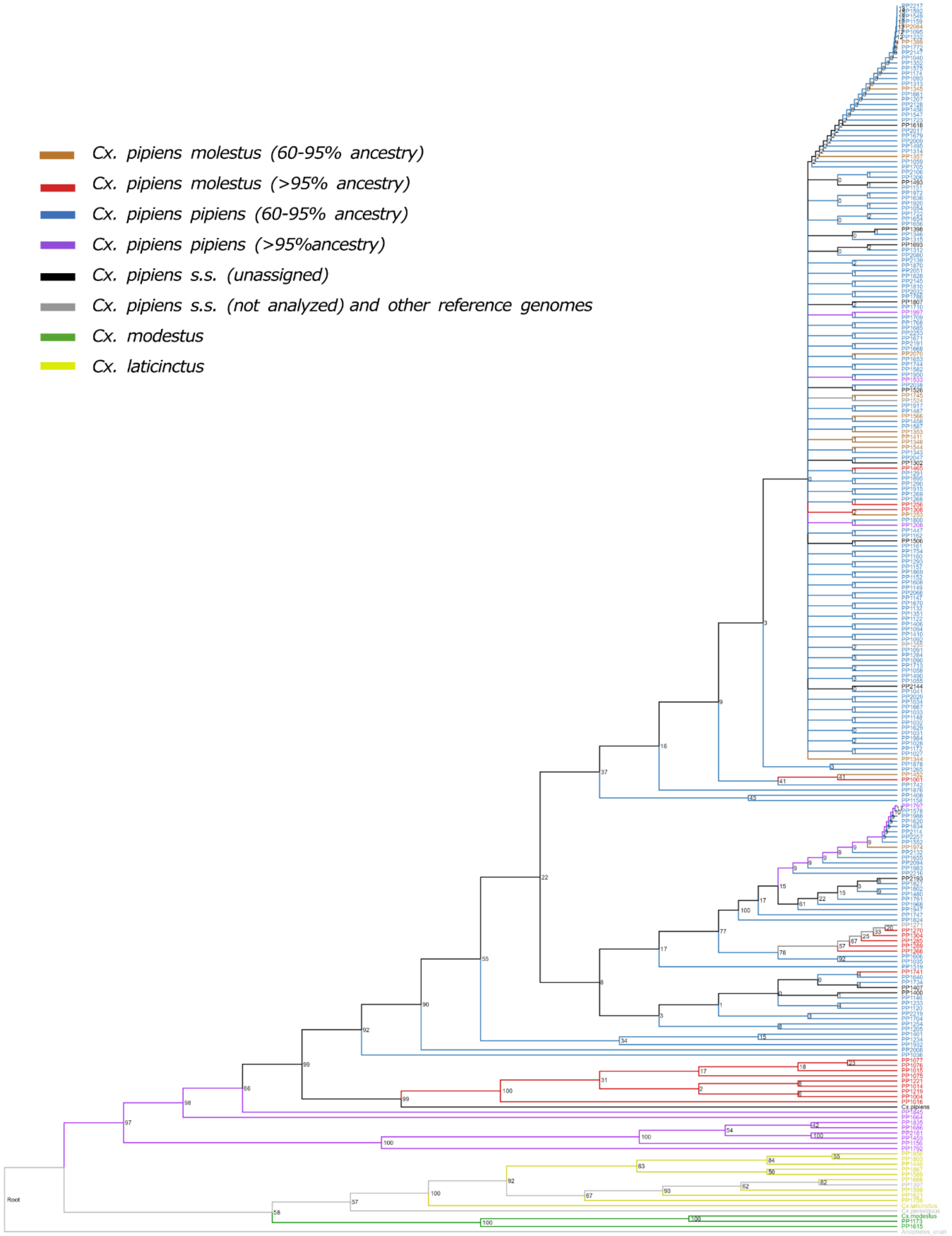

Supplementary Figure 1: Phylogenetic reconstruction based on mitochondrial DNA of 231 *Culex* mosquitoes from southern Spain and *Culex spp.* reference genomes. *Cx. p. pipiens* are colored in blue (60-95% *Cx. p. pipiens* ancestry) and purple (≥95% *Cx. p. pipiens* ancestry). *Cx. p. molestus* are colored in orange (60-95% *Cx. p. molestus* ancestry) and red (≥95% *Cx. p. molestus* ancestry). *Cx. pipiens s.s.* unassigned to ecotype (40-60% ecotype ancestries) and *Cx. p. pipiens* reference genome are shown in black. Ecotype assignments are based on nuclear DNA. Samples belonging to other *Culex* species, their reference genomes, and *Cx. pipiens s.s.* samples not included in further analyses because of high missing data are shown in grey.

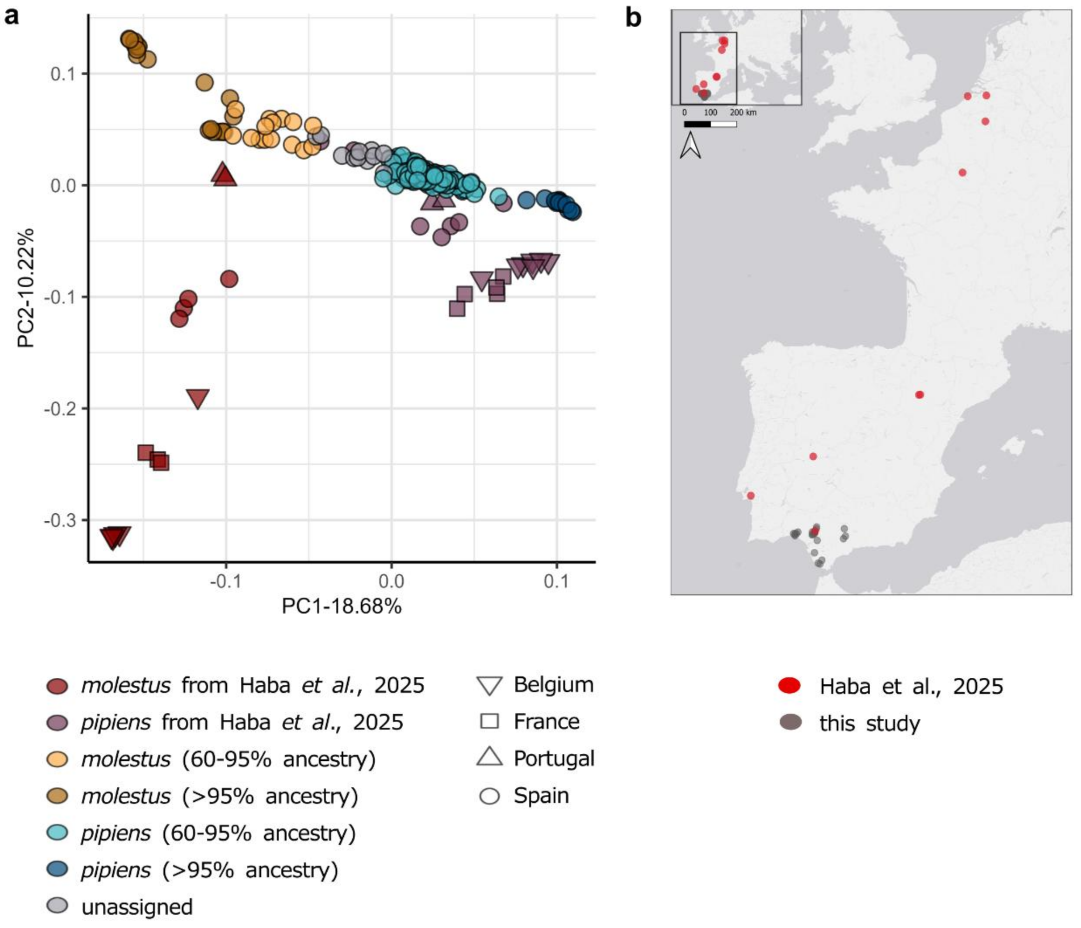

Supplementary Figure 2: Principal Components Analysis (PCA) including *Culex pipiens* from the present study (N=217) and from Haba *et al*. (2025, N=48). a) PCA results showing ecotype assignment. b) Map of trapping locations of samples included in the analyses.

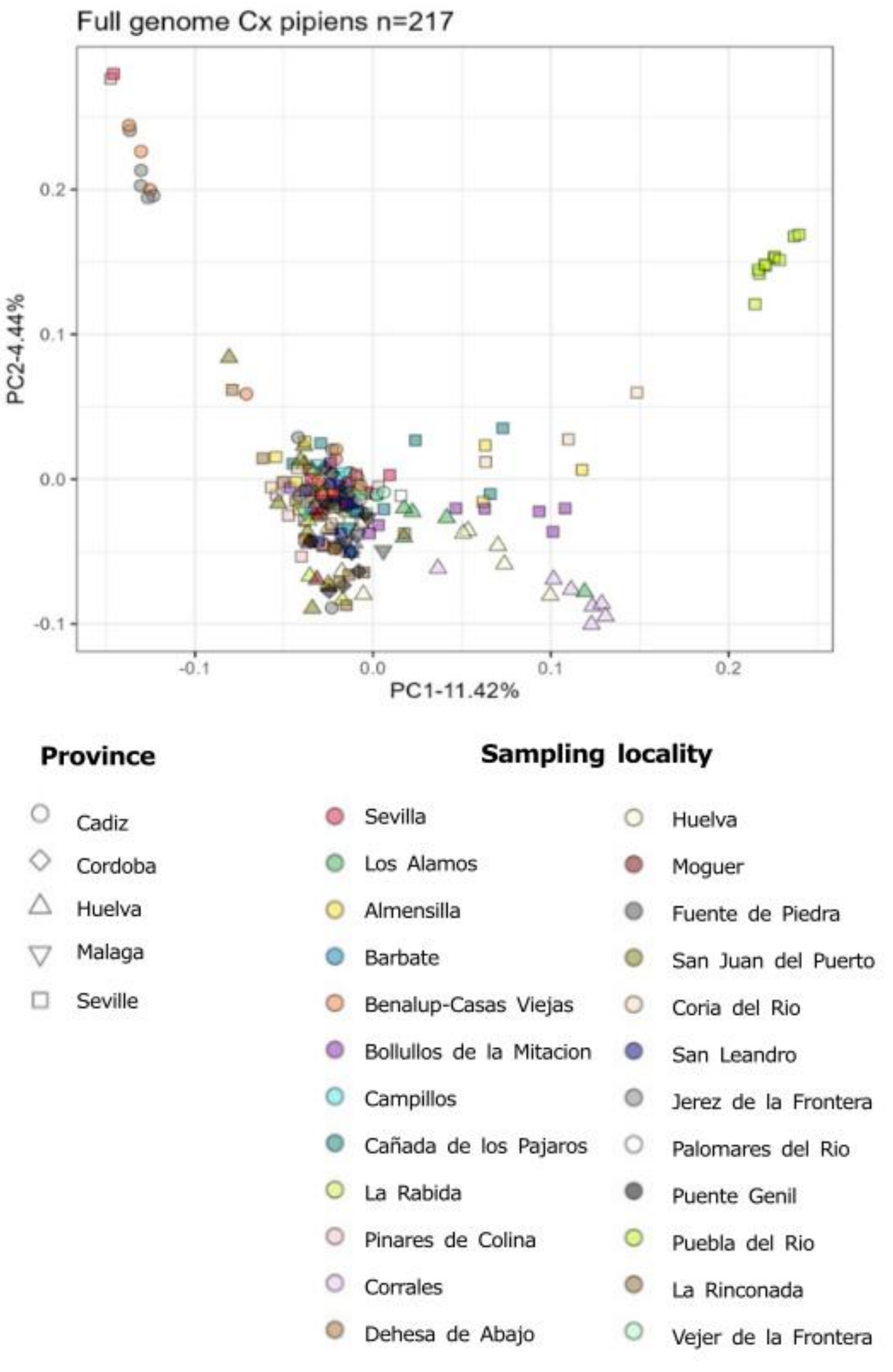

Supplementary Figure 3: Principal Components Analysis (PCA) of 217 *Culex pipiens* accounting for relatedness between samples (kinship threshold = 0.125).

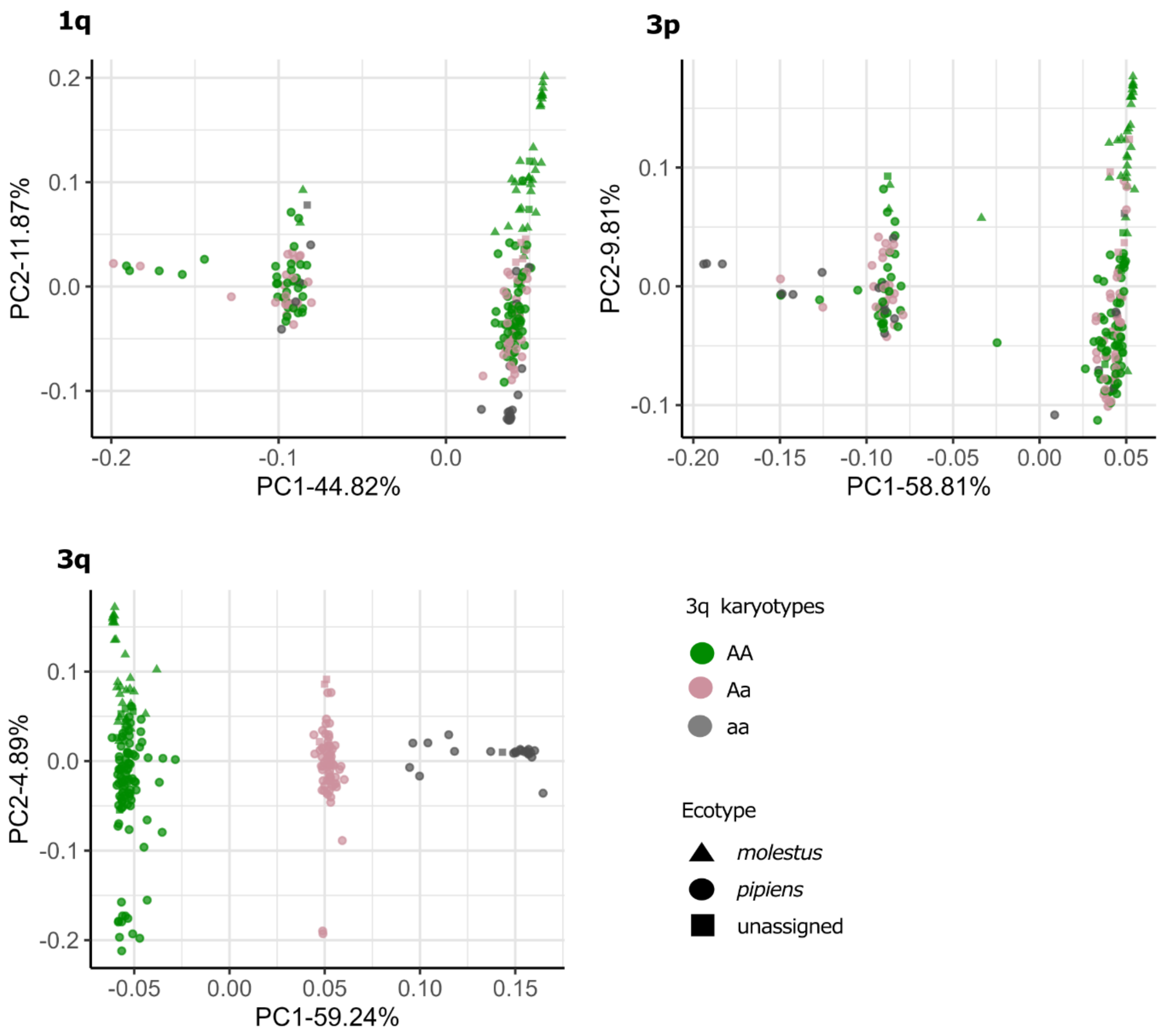

Supplementary Figure 4: Principal Components Analysis (PCA) of 217 *Culex pipiens* restricted to regions inside putative chromosomal inversions 1q (88-100Mbp, chromosome 1), 3p (4.8-17.5Mbp, chromosome 3) and 3q (150-160Mbp, chromosome 3). Samples are colored based on the karyotype they show in inversion 3q.

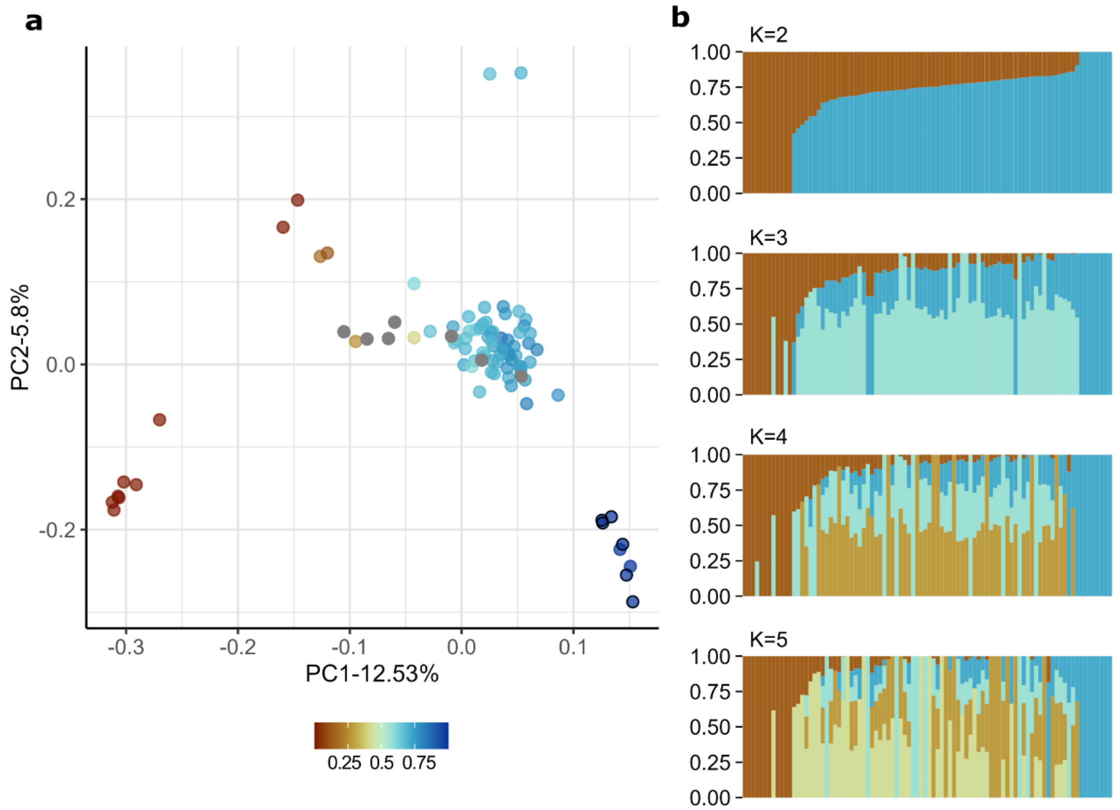

Supplementary Figure 5: Population structure analyses of *Culex pipiens* controlling for the confounding effect of chromosomal inversions and missing genotypes. a) Principal Components Analysis (PCA) of 90 *Cx. pipiens s.s.* Color gradient represents estimated proportion of *Cx. p. pipiens* ancestry (p(Q2) in ADMIXTURE analyses with K=2): samples with higher *Cx. p. pipiens* ancestry are shown in blue, whereas those with higher *Cx. p. molestus* ancestry are shown in orange. Grey dots represent samples that could not be confidently assigned to any of the ecotypes. Samples uninfected by Wolbachia are represented with a black outline. b) ADMIXTURE results for K=2 to K=5, illustrating individual ancestry proportions.

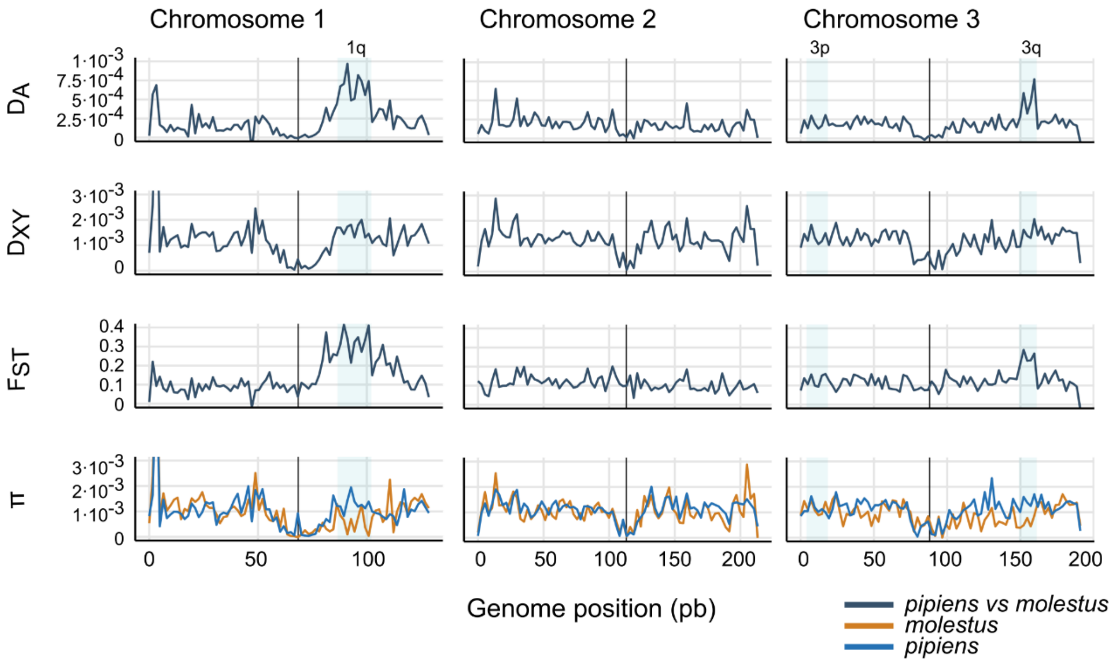

Supplementary Figure 6: D_{XY}, F_{ST} and D_A between *pipiens* and *molestus* and nucleotide diversity (π) estimated over 10kb genomic windows in specimens with 95% or higher ecotype ancestry. Centromeres are indicated with vertical black lines. Shade blue areas mark putative chromosomal inversions.

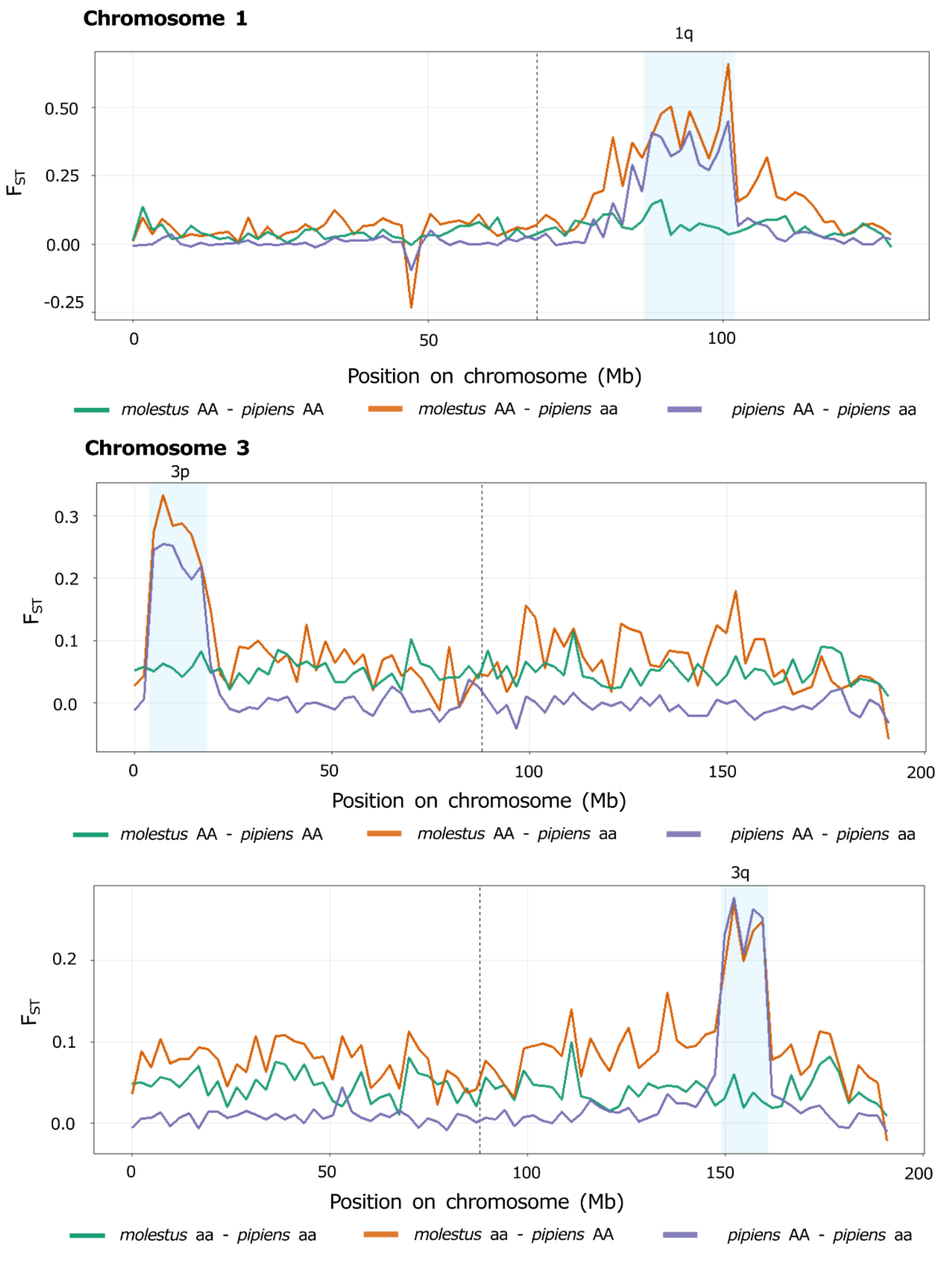

Supplementary Figure 7: F_{ST} between *pipiens* and *molestus* with standard (AA) and inverted (aa) karyotypes for the three putative chromosomal inversions. F_{ST} values were estimated over 10kb genomic windows using specimens with 60% or higher ecotype ancestry. Centromeres are indicated with vertical dashed lines. Shade blue areas mark putative chromosomal inversions.

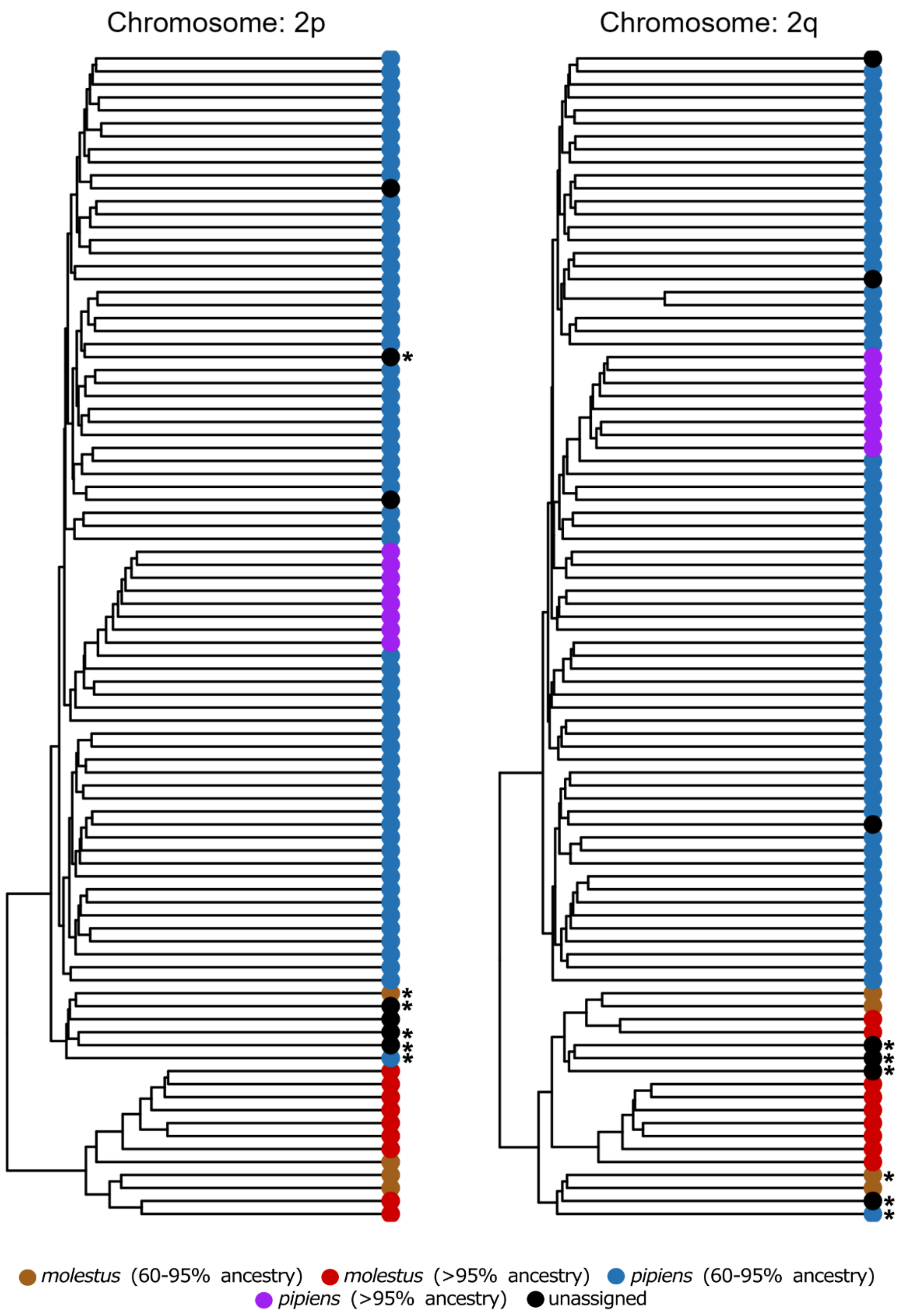

Supplementary Figure 8: Phylogenetic reconstruction of 90 *Culex pipiens* based on biallelic SNPs from both arms of chromosome 2. Samples are colored based on ecotype assignment. Samples * indicate samples with discordant lineage assignment between chromosome arms.

Supplementary Tables

Supplementary Table 1: Summary of *Culex pipiens* mosquitoes included in this study. Mosquitoes with >95% ecotype ancestry are shown in parenthesis. Habitat indicates if the sampling location was categorized as urban (U) or natural (N).

Province	Sampling location	X	Y	*molestus*	*pipiens*	unassigned	N total	Habitat
Cadiz	Barbate	36.2302	-5.8818	0	2	0	2	N
	Benalup-Casas Viejas	36.3567	-5.7933	0	9 (4)	0	9	N
	Jerez de la Frontera	36.614	-6.0588	0	9 (5)	0	9	N
	Vejer de la Frontera	36.2585	-5.9599	0	9	1	10	U
Cordoba	Puente Genil	37.3808	-4.7774	0	10	0	10	U
Huelva	Corrales	37.277	-6.9878	6 (6)	2	1	9	U
	Huelva	37.2422	-6.9274	5	4	0	9	U
	La Rabida	37.2051	-6.9262	0	10	0	10	U
	Los Alamos	37.2777	-6.9085	2	4	3	9	U
	Moguer	37.269	-6.8605	0	10	0	10	U
	San Juan del Puerto	37.3245	-6.795	0	9	0	9	U
Malaga	Campillos	37.0481	-4.8342	0	10	0	10	N
	Fuente de Piedra	37.1306	-4.7424	0	10	0	10	U
Seville	Almensilla	37.2994	-6.1008	1	6	2	9	U
	Bollullos	37.3151	-6.1685	5 (1)	5	0	10	U
	Cañada de los Pajaros	37.2389	-6.1289	2	5	2	9	N
	Coria del Rio	37.2828	-6.0666	3 (2)	5	0	8	U
	Dehesa de Abajo	37.2166	-6.1847	0	8 (1)	0	8	N
	La Rinconada	37.477	-5.9779	0	6	1	7	U
	Palomares del Rio	37.3158	-6.0566	0	9	1	10	U
	Pinares de Colina	37.2292	-6.1552	0	10 (1)	0	10	U
	Puebla del Rio	37.2596	-6.074	10 (10)	0	0	10	U
	San Leandro	37.0249	-5.9709	0	10	0	10	N
	Seville	37.4129	-5.9976	0	9 (1)	1	10	U
			**Total**	34 (19)	171 (12)	12	217	

Supplementary Table 2: Summary of *Culex pipiens pipiens* and *Culex pipiens molestus* samples from Haba *et al*. (2025) included in Principal Component Analyses (PCA) for ecotype assignment confirmation.

Country	Locality	SRA ID	Sample ID	Sex	Year	*Ecotype*
Belgium	Beveren	SRR31960279	BVR1	Female	2018	*molestus*
		SRR31960277	BVR2	Male	2018	*molestus*
		SRR31960276	BVR3	Female	2018	*molestus*
		SRR32271706	BVR4	Male	2018	*molestus*
		SRR32271705	BVR5	Female	2018	*pipiens*
		SRR32271704	BVR6	Female	2018	*pipiens*
		SRR32271703	BVR7	Female	2018	*pipiens*
	Frameries	SRR31960275; SRR32255677	FRM1	Female	2018	*pipiens*
		SRR32271711	FRM2	Female	2018	*pipiens*
		SRR32271710	FRM3	Female	2018	*pipiens*
		SRR32271709	FRM4	Male	2018	*pipiens*
	Zeebrugge	SRR31960283; SRR32255681	ZBRb1	Female	2017	*molestus*
		SRR31960281; SRR32255679	ZBRb3	Female	2017	*molestus*
		SRR31960280; SRR32255678	ZBRb4	Female	2017	*molestus*
		SRR32271682	ZBRb5	Female	2017	*molestus*
France	Paris	SRR32256194	PAR1	Male	2019	*pipiens*
		SRR32256193	PAR2	Female	2019	*pipiens*
		SRR32256158	PARb1	Female	2019	*molestus*
		SRR32256198	PARb2	Male	2019	*molestus*
		SRR32256196	PARb4	Male	2019	*molestus*
		SRR32256195	PARb5	Female	2019	*pipiens*
		SRR32271964	PARb6	Unknown	2019	*pipiens*
		SRR32271963	PARb7	Unknown	2019	*pipiens*
Portugal	Setubal	SRR32271840	BDA1	Male	2015	*pipiens*
		SRR32271839	BDA2	Female	2015	*molestus*
		SRR32271838	BDA3	Female	2015	*molestus*
		SRR32271834	BDA4	Female	2015	*molestus*
		SRR32271842	BDAi1	Female	2015	*molestus*
		SRR32271841	BDAi2	Female	2015	*pipiens*
		SRR32271836	BDAi3	Female	2015	*pipiens*
		SRR32271835	BDAi4	Female	2015	*molestus*
		SRR32271923	BDAi5	Female	2015	*molestus*
		SRR32271922	BDAi6	Female	2015	*molestus*
Spain	Extremadura	SRR32271823	EXT1	Female	2018	*pipiens*
	Garrapinillos	SRR32280606	GPN1	Female	2019	*pipiens*
		SRR32280605	GPN2	Female	2019	*pipiens*

	SRR32280604	GPN3	Female	2019	*molestus*
	SRR32271828	GPN4	Female	2019	*pipiens*
	SRR32271827	GPN5	Female	2019	*pipiens*
Zaragoza	SRR32269686	GPNi1	Female	2019	*molestus*
	SRR32269685	GPNi2	Female	2019	*molestus*
	SRR32271825	GPNi4	Female	2017	*pipiens*
	SRR32271824	GPNi5	Female	2017	*molestus*
Seville	SRR32271833	MDA1	Female	2019	*pipiens*
	SRR32271832	MDA2	Female	2019	*pipiens*
	SRR32271831	MDA3	Female	2019	*pipiens*
	SRR32271830	MDA4	Female	2019	*pipiens*
	SRR32271829	MDA5	Female	2019	*pipiens*

Supplementary Table 3: Karyotype frequencies estimated for three putative chromosomal inversions over 217 *Culex pipiens*. Mosquitoes with >95% ecotype ancestry are indicated in brackets. NA corresponds to mosquitoes in which karyotype assignment was not possible.

Inversion	Karyotype	*molestus*	*pipiens*	unassigned	Total
**1q**	wildtype	31 (19)	104	11	146
	heterokaryotype	2	54 (6)	1	57
	inverted		11 (6)		11
	NA	1	2		3
**3p**	wildtype	32 (19)	109 (12)	11	152
	heterokaryotype	2	54	1	57
	inverted		7		7
	NA		1		
**3q**	wildtype		21 (12)	1	22
	heterokaryotype		63	4	67
	inverted	34 (19)	87	7	128

Supplementary Table 4: Gene Ontology (GO) enrichment analysis restricted to chromosomal inversions. Only GO terms significant at α = 0.05 after false discovery rate (FDR) correction are shown.

Inversion	GO Term	Function	Category	Count	Total genes	p-value	FDR
3p	GO:0050911	detection of chemical stimulus involved in sensory perception of smell	BP	14	103	1.70E-07	7.06E-04
	GO:0050896	response to stimulus	BP	53	1344	2.60E-07	7.06E-04
	GO:0140359	ABC-type transporter activity	MF	14	55	2.90E-10	5.77E-07
	GO:0020037	heme binding	MF	20	181	2.30E-07	2.29E-04
	GO:0004984	olfactory receptor activity	MF	14	103	1.30E-06	8.61E-04
	GO:0005506	iron ion binding	MF	19	199	4.30E-06	2.14E-03
	GO:0016705	oxidoreductase activity, acting on paired donors, with incorporation or reduction of molecular oxygen	MF	19	205	6.70E-06	2.39E-03
	GO:0004497	monooxygenase activity	MF	19	206	7.20E-06	2.39E-03
	GO:0016887	ATP hydrolysis activity	MF	20	229	9.40E-06	2.67E-03
	GO:0005789	endoplasmic reticulum membrane	CC	23	295	1.00E-05	9.39E-03
3q	GO:0017121	plasma membrane phospholipid scrambling	BP	5	7	3.90E-08	2.12E-04
	GO:0042302	structural constituent of cuticle	MF	16	123	7.80E-09	1.55E-05
	GO:0016747	acyltransferase activity, transferring groups other than amino-acyl groups	MF	16	158	6.90E-08	6.03E-05
	GO:0017128	phospholipid scramblase activity	MF	5	7	9.10E-08	6.03E-05
	GO:0005212	structural constituent of eye lens	MF	3	3	9.90E-06	4.92E-03

	GO:0008609	alkylglycerone-phosphate synthase activity	MF	3	5	9.60E-05	3.82E-02
	GO:0045277	respiratory chain complex IV	CC	3	4	1.80E-05	1.69E-02
1q	GO:0006520	amino acid metabolic process	BP	10	153	5.00E-07	2.71E-03

Supplementary Table 5: f3 statistics for three different combinations of source and target populations between *Culex pipiens pipiens*, *Culex pipiens molestus* and samples of *Culex pipiens* unassigned to any of the ecotypes. Significant results indicating signs of admixture are shown in bold (|Z| ≥ 3).

Target	Source1	Source2	f_3	std.err	Z
unassigned (N=7)	*molestus*	*pipiens*	-9.32E-04	3.78E-04	-2.467
molestus (N=13)	*pipiens*	unassigned	6.01E-04	2.19E-04	2.746
**pipiens (N=70)**	**unassigned**	***molestus***	**-5.83E-04**	**1.68E-04**	**-3.46**

Supplementary Table 6: Reference *pk1* sequences from different *w*Pip groups used to classify *w*Pip consensus assemblies from 217 *Culex pipiens.*

Accession number	*Strain*	Reference
OM885005.1	wPip-I	(Atyame *at al.*, 2011)
OM885006.1	wPip-III	(Atyame *at al.*, 2011)
OM885007.1	wPip-II	(Atyame *at al.*, 2011)
OM885008.1	wPip-IV	(Atyame *at al.*, 2011)
OM885009.1	wPip-V	(Atyame *at al.*, 2011)
LC106155.1	wPip-I	(de Pinho Mixão *et al*., 2016)
OQ223315.1	wPip-II	(da Moura *et al*., 2023)
OQ223310.1	wPip-III	(da Moura *et al*., 2023)
OQ223309.1	wPip-IV	(da Moura *et al*., 2023)
